# Spread of Carbapenem-Resistant *Klebsiella pneumoniae* in Hub and Spoke Connected Health-Care Networks: A Case Study from Italy

**DOI:** 10.3390/microorganisms8010037

**Published:** 2019-12-23

**Authors:** Pamela Barbadoro, Arianna Dichiara, Daniele Arsego, Elisa Ponzio, Sandra Savini, Esther Manso, Marcello M. D’Errico

**Affiliations:** 1 Dipartimento Scienze Biomediche e Sanità Pubblica, Facoltà di Medicina e Chirurgia, Università Politecnica delle Marche, 60122 Ancona, Italy; p.barbadoro@staff.univpm.it (P.B.); arianna.dichiara@libero.it (A.D.); danielearsego@gmail.com (D.A.); elisaponzio@staff.univpm.it (E.P.); 2 SOD Igiene Ospedaliera, AOU, Ospedali Riuniti, 60126 Ancona, Italy; sandra.savini@ospedaliriunit.marche.it; 3 SOS Microbiologia, AOU, Ospedali Riuniti, 60126 Ancona, Italy; e.manso@ospedaliriuniti.marche.it

**Keywords:** healthcare-associated infections, antimicrobial resistance, automated surveillance, infection control

## Abstract

The study describes the spread of carbapenem-resistant *Klebsiella pneumoniae* (CRKP) in a regional healthcare network in Italy. The project included several stages: (1) Establishment of a laboratory-based regional surveillance network, including all the acute care hospitals of the Marches Region (*n* = 20). (2) Adoption of a shared protocol for the surveillance of Multi-Drug Resistant Organisms (MDROs). Only the first CRKP isolate for each patient has been included in the surveillance in each hospital. The anonymous tracking of patients, and their subsequent microbial records within the hospital network, allowed detection of networks of inter-hospital exchange of CRKP and its comparison with transfer of patients within the hospital network. Pulsed-Field Gel Electrophoresis (PFGE) analysis has been used to study selected isolates belonging to different hospitals. 371,037 admitted patients have been included in the surveillance system. CRKP has shown an overall incidence rate of 41.0 per 100,000 days of stay (95% confidence interval, CI 38.5–43.5/100,000 DOS), a CRKP incidence rate of isolation in blood of 2.46/100,000 days of stay (95% CI 1.89–3.17/100,000 days of stay (DOS) has been registered; significant variability has been registered in facilities providing different levels of care. The network of CRKP patients’ exchange was correlated to that of the healthcare organization, with some inequalities and the identification of bridges in CRKP transfers. More than 73% of isolates were closely related. Patients’ exchange was an important route of spread of antimicrobial resistance, highlighting the pivotal role played by the hub, and selected institution to be used in prioritizing infection control efforts.

## 1. Introduction

The incidence of Multi-Drug Resistant Organisms (MDROs) has dramatically increased over the last decades and is now considered one of the preeminent infectious disease problems in Europe, and worldwide [1]. Data obtained from national experiences and those obtained by the European Survey on Carbapenemase-Producing Enterobacteriaceae (EuSCAPE) have shown how Italy is among the European countries with an higher burden of disease caused by Carbapenem Resistant Enterobacteriaceae (CRE) [2,3,4,5,6]. Data from the Italian surveillance of antibiotic resistance reporting to the European Antimicrobial Resistance Network (EARS-Net), showed that from a non-susceptibility of 1.3%; in 2009, the percentage of carbapenem-resistant *Klebsiella pneumoniae* (CRKP) causing BSI increased dramatically to 34.3% in 2013, and has now established at 26.8% in 2018 making *Klebsiella pneumoniae* the most important antibiotic-resistance threat in Italy [7]. The importance of acute care hospital organization, management, and structure for prevention of healthcare-associated infections has been previously remarked [8]. In addition to locally-based infection control policies, the ongoing reorganization of healthcare providers within a regionally managed healthcare service based on network models occurring in the Marches region of Italy, may have an impact in infection control; in fact, it has been already shown that patients’ transfers may act as pivotal routes of transmission [9,10,11], and highly connected hospitals may have the most potential to transmit MDROs in the entire network [10,12,13]. Furthermore, patients with a positive clinical culture result are at higher risk of readmission, may sustain the spread of MDROs within the network and should indeed be targeted to receive additional discharge planning resources that could be useful also in the context of infection control [14]. Surveillance of antibiotic resistance at a regional level is an essential component of infection control [15,16] and, indeed, the adoption of regional infection control measures has been already associated to the reduction in incidence rates of spread over time [8,17,18]. The current CRE control policy in Italy is based on active screening in all contacts of CRE-positive patients, in all patients with a previous colonization/infection that are admitted to hospital, and in all patients coming from endemic areas. Moreover, screening has been suggested for patients admitted or transferred to high-risk units and in patients transferred from another hospital or with a history of recent hospitalization/stay in long term care facilities [19,20]. Screening on admission represents an important activity of local microbiology laboratory and the crucial support of laboratory information in infection control may be improved by the analysis of patient movements within the network, in order to highlight high-risk routes of transmission.

Within an interregional project supported by the Italian National Centre for Disease Prevention and Control (CCM), the aim of this paper is to model the spread of carbapenem-resistant *Klebsiella pneumoniae* (CRKP), as well as patients’ movements between hospitals, in the regional healthcare network of the Marches Region to define priorities for infection control.

## 2. Materials and Methods

**Figure 1:**
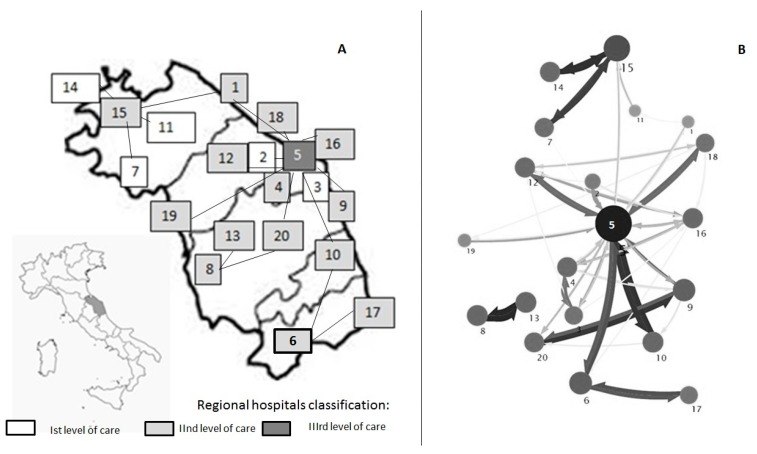
Distribution of hospitals according to level of care, and reciprocal hub-and-spoke connections (**A**), and mapping of inter-facility transfer of CRKP isolates within the Marches hospital network (**B**). (Marches region, Italy, 2014). Each hospital structure is represented by a grey circle. The diameter of the circle is proportional to the amount of MDROs exchanged. The color intensity and the size of the arrows represents the number of CRKP exchanged between hospitals. The direction of the arrows represents the direction of the exchanges. The map has been realized using the Mapequation software (http://www.mapequation.org/)

The Marche region, located on the eastern coast of Italy, has a population of about 1.5 million inhabitants. Hospitals have been defined has follows: 1st level (primary care, district hospital or first-level referral, with mainly internal medicine, obstetrics-gynecology, pediatrics, general surgery wards), 2nd level (highly differentiated wards with at least some clinical specialties, including intensive care units); 3rd level (tertiary care, hospital with teaching function, and regional reference). The local hospital-level organization is based on the hub-and-spoke design, with different (*n* = 5) 1st level hospitals referring to 2nd level nodes (*n* = 14), and thus conveying on a regional tertiary care institution based in Ancona (Figure 1A). The above healthcare organization involves the frequent exchange of patients between hospitals, and, therefore has stimulated the interest for a regional approach in surveillance of antibiotic resistance. The project included several stages:

(1) Establishment of a laboratory-based regional surveillance network, coordinated by the Hospital Hygiene Unit of the Azienda Ospedaliero-Universitaria Ospedali Riuniti di Ancona (university, tertiary care institution), including all the acute care hospitals of the Marches region. Definition of a regional data integration system (based on the local Laboratory Information System, and standardizing antibiotic panels testing, sampling site definition, as well as microorganism codification). Participants to the network (MICA-net) at the time of this survey are listed in Appendix A. Anonymization, but univocal encoding, of patients colonized/infected by CRKP, to trace the subsequent isolates from the same patient in different hospitals of the region. All data from invasive and non-invasive isolates have been included in the surveillance system. Sampling sites have been summarized and re-coded as: airways, blood, surgical wound, urine, and other sites. Isolates were obtained from clinical specimens submitted for diagnostic purposes; samples obtained for screening were excluded from this analysis. Statistical significance of differences in the proportion of anatomical sampling detection sites between carbapenem-sensitive *Klebsiella pneumoniae* (CSKP) and CRKP were evaluated by chi-squared test. Analyses were performed with STATA, release 15.1 (StataCorp LLC., College Station, TX, USA).

(2) *Klebsiella pneumoniae* isolates have been classified as carbapenem-resistant based on international guidelines [21]. Only the first CRKP isolate for each patient, in each participating hospital, has been included in the surveillance. Incidence rate has been calculated by dividing the number of first isolates (not including patients already positive in other settings)/100,000 patient days; CRKP-bloodstream infections (CRKP-BSI) have been defined as those occurring in patients with at least one positive blood culture (only the first isolate has been included, therefore organism identified in blood were not related to an infection at another site). CRKP-BSI rates across different levels of care (i.e., I, II, III) and areas (medical, critical and surgical care) have been calculated per 100,000 days of hospital stay. Resistance rate has been detailed for blood specimens. For metric purposes, only the first MDRO isolate recovered from a patient during a given surveillance period has been included, so that the rates of MDRO colonization or infection are not overestimated. [22]

(3) Detection and visualization of networks of inter-hospital CRKP exchange (MDROs network) have been performed using the Infomap software [23]. Network analysis, in this case, has been used to capture the flow of microorganisms between the nodes of the healthcare system (i.e., hospitals), and to find functional communities consisting of nodes exchanging CRKP. For this objective, the isolation of the same CRKP (at the gene/species antibiotic resistance pattern) in the patient exchanged between different institutions has been considered.

In order to calculate network structure connectivity metrics, we accessed the regional Hospital Discharge Records (HDR) database, analyzing all pairs of movements of patients among healthcare facilities in the 2013–2014 period. The movements have been recorded in terms of “direct” and “indirect transfers” (including up to one year of stay in the outpatient setting) [24].

Network analysis was carried out to examine the previously-defined two networks of HDR, and MDROs. Network connectedness was calculated including density rate (indicating the degree to which the entities in a network are connected), ranging from zero (completely unconnected) to one (completely connected), reciprocity (likelihood of entities in a directed network to be mutually linked the proportion of mutually connected dyads overall connected dyads, transitivity (or clustering), in-degree (i.e., total number of patients received at a hospital from all others), weighted out-degree, betweenness centralization (is an indicator of an hospital’s influence in a network) and centrality (Closeness centrality is higher centrality scores to vertices that are situated closer to members of their component, or the set of reachable vertices, by taking the inverse of the average shortest paths as a measure of proximity) [25]. Moreover, the presence of bridges between nodes of the hospital as well as MDROs networks and between network correlations have been assessed. Multiple Regression Quadratic Assignment Procedure (QAP) has been used to study the relation between CRKP-BSI taking into account the network structure of the HDR, and level of care. Network analysis has been carried out by using the “nwcommands” package in Stata version 15.1 SE [26,27].

(4) Pulsed Field Gel Electrophoresis (PFGE) has been used to evaluate clonal relatedness of CRKP isolates randomly selected in different hospital laboratories participating in the network (*N* = 15). DNA was prepared in agarose plugs and digested with 20 U XbaI restriction enzyme (Invitrogen, Leek, The Netherlands); PFGE was performed using a CHEF Mapper (CHEF DR II System, Bio-Rad, Hercules, CA, USA), with a run time of 24 h at 14 °C, using switch times of 10–45 s. The gel was stained with ethidium bromide (10 mg/mL) and photographed under UV light. Conventional procedures and criteria have been used for interpretation [28].

Ethics Approval and Consent to Participate: Administrative permission to access laboratory data has been obtained by all Medical Directors of participating laboratories and hospitals. Analyzed data had been automatically de-identified by the software house managing the Regional Laboratory Information System by means of a casual algorithm composed of 30 characters (i.e., PIW3OSKKNIMOOCK8NKBVWVNCW1XEWQO). Since the resistant isolates were collected from patients’ routine samples, and that patients’ data have been previously anonymized and retrieved from the laboratory information system, this study was considered as a laboratory study and ethics approval from institutional ethics committee was not required.

## 3. Results

**Figure 2:**
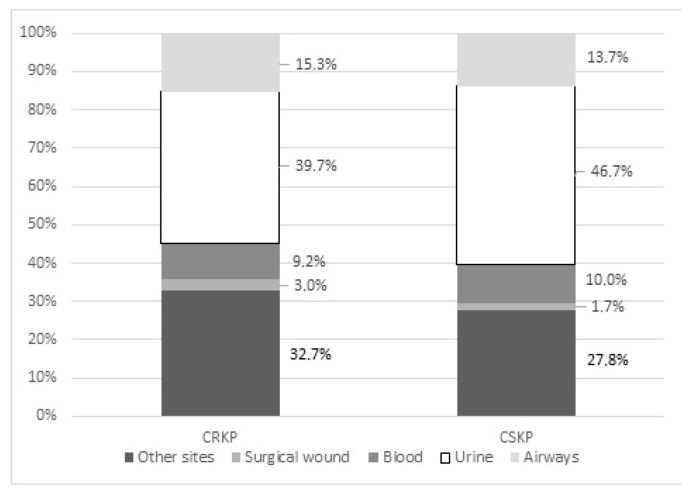
Distribution of sampling sites in carbapenem-resistant (CRKP) versus carbapenem sensitive *Klebsiella pneumoniae (CSKP) strains.* (Marches region, Italy, 2014).

In the January 2013–December 2014 period, 371,037 admitted patients have been included in the surveillance system. About 38.7% of *Klebsiella pneumoniae* first isolates were resistant to carbapenems; carbapenem resistance rate in isolates from blood was 29.4%. CRKP has shown an overall incidence rate of 41.0 per 100,000 days of stay (95% CI 38.5–43.5/100,000 DOS). The most frequent sampling site for CRKP was the urinary tract (39.7% of strains), followed by surgical wounds (32.7% of cases), and 9.2% of isolates belonged to blood samples (Figure 2). In the study period a CRKP incidence rate of isolation in blood of 2.46/100,000 days of stay (95% confidence interval, CI 1.89–3.17/100,000 days of stay, DOS) has been registered, with levels ranging between 1.30/100,000 days of stay (95% CI 0.27–3.81/100,000 DOS) in first level hospitals to 2.07/100,000 (95% CI, 1.42–2.93/100,000) in second level facilities, up to 3.66/100,000 days of stay (95% CI, 2.41–5.32/100,000 DOS) in third level facilities; dealing with data belonging to selected clinical areas, 1.17 cases/100,000 DOS (95% CI, 0.53–2.21/100,000) have been registered in surgical areas; intermediate risk has been measured in medical wards with 2.81/100,000 DOS (95% CI 2.03–3.78/100,000 DOS) while critical areas have registered higher rates (4.84/100,000 DOS, 95% CI 2.32–8.90/100,000).

Detection of subsequent isolation of CRKP in colonized/infected patients in the regional acute care network (*n* = 260) has allowed the identification of major routes within the network itself; the resulting referral pattern was consistent with the theoretical organization of hospitals in the Region (Figure 1A), pointing out the priorities for action and sub-areas of interventions in selected hospitals (Figure 1B).

**Figure 3:**
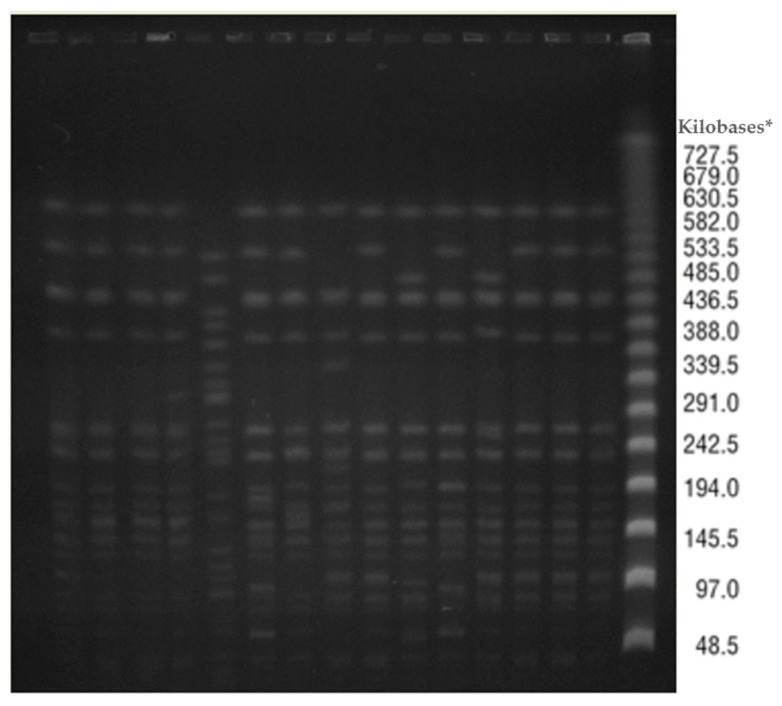
Distribution of PFGE patterns of selected random isolates circulating in the regional healthcare network. (Marches region, Italy, 2014). * As reference standard a Lambda Ladder PFG marker was used (NewEngland BioLabs, Ipswich, MA, USA).

PFGE has highlighted indistinguishable strains in two-thirds of selected isolates (*n* = 10), three closely related strains with only one or two bands of difference, plus one unrelated isolate (Figure 3).

**Figure 4:**
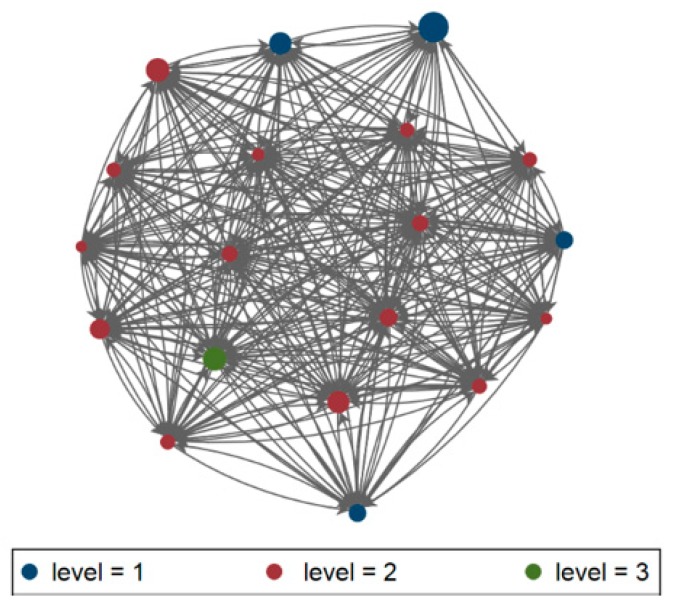
Visualization of the Marches HDR Network, 2013–2014. Node colour corresponds to facility type (I, II, III level of care), and size of node corresponds to number of admissions.

**Figure 5:**
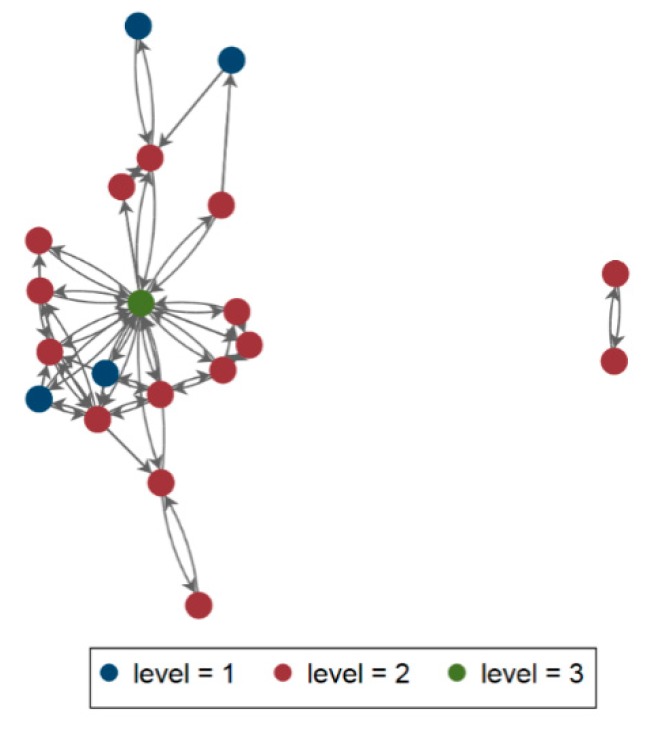
Visualization of the Marches MDRO network, 2013–2014. Node colour corresponds to facility type (I, II, III level of care), and size of node corresponds to number of exchanged MDROs.

The patient sharing network as defined by the Regional Hospital Discharge Records (HDR) consisted of 20 facilities. The visualization of the patient transfer-defined regional healthcare network (Figure 4) has defined a network including 20 nodes and 306 arcs, exchanging a minimun of zero and a maximum of 826 patients, with a density rate of 0.81 (thus indicating that hospitals in this network are quite connected), reciprocity of 0.83 (hospitals exchange multiple patients), transitivity (or clustering) of 0.50, betweenness centralization of 0.004 (indicating that hospitals in this network are quite decentralized), indegree centralization of 0.15 and outdegree of 0.15; on the other hand, the MDROs exchange network (Figure 5) has highlighted 59 arcs, with a maximum value of 19 patients, density of 0.16 reciprocity of 0.69 (both indicating a small amount of exchanges), transitivity (or clustering) of 0.14, betweenness of 0.60 indegree centralization of 0.50 and outdegree centralization of 0.61. Bridges were not identified in the HDR network, while it has been possible the identification of multiple bridges linking hospitals within the MDROs network. The visualization of the CRKP case transfer network was overlaid on the Medicare transfer defined regional healthcare network (Figure 4 and Figure 5), and the two were indeed correlated by a significant (*p* < 0.05) degree of 0.59, at network analysis.

QAP analysis has confirmed the significant role of the HDR network (Coef. 0.01; 95% CI, 0.01–0.14), and level of care (Coef. −0.66; 95% CI, −1.31–−0.01) in CRKP rate (the adjusted R2 of the QAP regression analysis was 0.219, *p* < 0.001).

## 4. Discussion

The present project has led to the establishment of a surveillance network for the monitoring of the spread of MDROs within the Marches region, and between different hospitals. The overall incidence rate of CRKP has been higher with respect to that estimated for Italy in the context of the European wide EuScape survey [2], but in line with those previously reported in Italy as well as in other settings [18,29,30]. Bloodstream infection rates are in line with those recently published from the Italian national surveillance system, reporting that BSI rate by carbapenem-resistant Enterobacteriacee among hospitalised patients was 2.31 per 100,000 in-habitants in 2014 in Italy [31]. The incidence rate of BSI was higher in Intensive Care Units of the Marches region, reaching 4.84/100,000 DOS (95% CI 2.32–8.90/100,000) in critical areas, which is slightly lower than the peak of 17.7/100,000 registered in the same year in the Liguria region of Italy [32], confirming the importance of critical areas in the development of CRKP-BSI [33]. Moreover, significant differences across the various levels of care have been measured, and should guide us towards a more in-depth analysis of local facilities; this results support the need of detailed surveillance data; as already underlined, policies should be tailored to local resistance rates, at least at the hospital level, and possibly with finer resolution [34]. The participation of intermediate/rehabilitation level facilities to the surveillance network will allow the analysis of the role and interactions of different levels of care in MDROs spread, and the lack of such information represent a limit of the present study.

The analysis of the exchanges of patients positive for CRKP within the hospital network has traced routes that overlap the theoretical design of the hub and spoke reference model of the hospital care provided in our region, and a significant correlation between the two networks, confirming, once more, the functional linkage, and potential for micro-organisms spread within the healthcare network [5,6]. Besides a functional one, a microbiological network has been established in the Marches region, and this important route of transmission needs to be controlled. The results of network analysis have suggested different behaviors within the HDR and the MDROS’ networks; in fact the HDR network has shown the exchange of a great number of patients, but with low values in centrality measures; on the other hand, the number of CRKP cases transferred between any two facilities (represented by edges, or lines in the figure) was obviously lower with respect to the number of patients, but centralization of MDROs network was higher, highlighting a well-organized network exchanging CRKP positive patients. These pathways within the MDRO network have been highlighted from the presence of bridges at network analysis; such bridges are even more interesting when compared to the apparent absence of them in the HDR network. From an infection control point of view, these findings support the usefulness of network analysis in defining priorities for action to limit the spread of antimicrobial resistance, especially dealing with newly emerging pathogens/mechanisms of resistance [12,35]. Despite the evidence of a small number of exchanged patients colonized/infected by MDROs with respect to the number of overall exchanged patients, the identification of specific patterns of movements of patients within the network supports the use of screening on admission policies [19] and highlights the opportunity of network analysis of patients’ movements in identifying specific high-risk routes as targets for focusing infection control (i.e.,: screening on admission policies) [19]. The importance of acute care hospital organization, management and structure for prevention of healthcare-associated infections has been previously remarked, and the adoption of regional infection control measures have led to a reduction in incidence rates of spread over time [8,9,10]. Patients’ exchange was an important route of spread of antimicrobial resistance, highlighting the pivotal role played by the hub as the most important actor in directing and organizing inter-hospital, and multi-level network efforts in infection control. The results have also highlighted the need for the introduction of more advanced molecular epidemiology technique linking genetic relatedness of strains to trace epidemiologically verified associations and to highlight eventual unidentified sources/routes of transmissions, especially in the outbreak setting [36]. In fact, the spread of genetically related strains in the whole region might confound epidemiological linkage, leading to potential mistakes in the context of an outbreak investigation. On the other hand, as evidenced by Donker et al. [37]; the isolation of strains of apparently unknown genomic linkage, may constitute an indication of a mostly undefined extra-institutional reservoir, connecting the hospital to the community, as well as the environmental and veterinary public health sectors.

## 5. Conclusions

The presented data confirm the importance of a comprehensive approach to antibiotic resistance in the regional context, because of the complexity of care. Further developments of the initiative include the adoption of a shared antibiotic policy, together with the routine utilization of genetic epidemiology both in the hospital, in the community, and at the human-animal-environment interface.

## Appendix A

MICA-NET: Francesca Orecchioni ^3;^ Enrica Martini ^3^, Donatella Mengoni ^3^, Maria Grazia Gioia ^3^, Rossella Stoico ^3^, Luca Formenti ^3^, Aurora Luciani ^3^, Rebecca Micheletti ^3^, Giovanni Tassinari ^4^; Barbara Pieretti ^4^; Patrizia Lobati ^4^; Nadia Storti ^5^; Gianluca Serafini ^5^; Vania Carignani ^5^; Olimpia Veccia ^5^; Fabrizio Barontini ^5^, Alessia Baldassarri ^6^; Romanini Ivana ^6^; Andrea Cani ^6^; Francesca Ermeti ^6^; Maria Elisabetta Pertosa ^6^; Gianni Aloisi ^6^; Enzo Pazzaglia ^6^; Ivana Sbaffi ^6^; Anna Maria Priori ^6^; Valeria Benigni ^6^, Simone Barocci ^6^; Stefano Lodolini ^6^; Emma Maurizi ^6^, Antonio Setaro ^6^; Virginia Fedeli ^6^; Sonia Bacelli ^6^; Paola Pauri ^6^; Emily Tili ^6^; Andrea Monachesi ^6^; Nicoletta Damiani ^6^; Annamaria Bellanova ^6^; Mauro Bassano ^6^; Giuseppina D’Amato ^6^; Luciana Gironacci ^6^; Eleonora Berardinelli ^6^; Marisiana Granatelli ^6^; Salvatore Licitra ^6^; Archedora Gasparrini ^6^; Patrizia Olori ^6^; Remo Appignanesi ^6^; Valeria Travaglini ^6^, Serenella David ^7^, Letizia Ferrara ^7^, Daniela Vincitorio ^7^; Roberta Galeazzi ^7^;Massimo Frascarello ^8^; Ketty Bojanovski ^8^; Antonio Macellari ^8^; Paolo Rocchi ^8^; Anna Marigliano ^1^, Francesco Labricciosa ^1^; Giorgia Mazzarini ^1^; Claudia Recanatini ^1^ and Federico Tirabassi ^1^

^4^ AO Ospedali Marche Nord, 61121 Pesaro, Italy; musa@univpm.it

^5^ AOU Ospedali Riuniti Ancona, 60126 Ancona, Italy

^6^ Azienda Sanitaria Unica Regionale, ASUR MARCHE, 60122 Ancona, Italy

^7^ INRCA, Italian National Institute for Aging, Ancona Hospital, 60127 Ancona, Italy

^8^ Istituto di Riabilitazione Santo Stefano, 62018 Porto Potenza Picena, Macerata, Italy
